# Research progress of extracellular vesicles derived from mesenchymal stem cells in the treatment of neurodegenerative diseases

**DOI:** 10.3389/fimmu.2025.1496304

**Published:** 2025-04-02

**Authors:** Haibin Shen, Jie Chen, Meijin Liu, Minghong Zhao, Die Hu, Fangfang Xie, Qing Jin, Dewang Xiao, Zongbo Peng, Tao Qin, Dingyu Rao, Defa Huang

**Affiliations:** ^1^ Laboratory Medicine, First Affiliated Hospital of Gannan Medical University, Ganzhou, China; ^2^ Department of Laboratory Medicine, Yongchuan Hospital of Chongqing Medical University, Chongqing, Yongchuan, China; ^3^ Laboratory Medicine, People’s Hospital of Ganzhou Economic Development Zone, Ganzhou, China; ^4^ Laboratory Medicine, Guizhou Aerospace Hospital, Zunyi, China; ^5^ The First School of Clinical Medicine, Gannan Medical University, Ganzhou, China; ^6^ Department of Cardiothoracic Surgery, The First Affiliated Hospital of Gannan Medical University, Ganzhou, China

**Keywords:** mesenchymal stem cells, extracellular vesicles, neurodegenerative disease, treatment, diagnosis

## Abstract

As the world’s population ages, neurodegenerative diseases are becoming more widely acknowledged as serious global health and socioeconomic issues. Although many resources have been devoted to the research of these illnesses, little progress has been made in the creation of novel diagnostic and therapeutic approaches. Extracellular vesicles (EVs) are released by all cell types and contain proteins, microRNAs, mRNAs, and other biologically active molecules. EVs play an important role in intercellular communication as well as in the regulation of neuroinflammation. Determining the mechanisms by which EVs contribute to the pathogenesis of neurodegenerative diseases will aid in the development of new therapeutic approaches and diagnostic tools. Mesenchymal stem cells (MSCs) have been shown in studies to control immunological responses, promote the growth of new brain connections, promote the production of blood vessels, and heal damaged tissues. There is growing evidence that MSCs’ ability to treat patients is mostly due to the neurotrophic compounds they secrete through EVs. Since their tiny size allows them to pass through biological barriers and reach injured parts of the central nervous system, MSC-derived extracellular vesicles (MSC-EVs) retain many of the therapeutic qualities of their parent MSCs. This review discusses the role of EVs in neurodegenerative diseases and highlights the potential of MSC-EVs in the treatment of neurodegenerative diseases. The paper also examines the challenges that still need to be overcome and the prospects for using MSC-EVs to treat neurodegenerative illnesses.

## Introduction

Neurodegenerative conditions are characterized by a gradual decline in neurons within both the central and peripheral nervous systems, leading to impaired motor and cognitive abilities (1). This group of disorders predominantly comprises Alzheimer’s disease (AD), Parkinson’s disease (PD), amyotrophic lateral sclerosis (ALS), and Huntington’s disease (HD) (2, 3). A primary pathological hallmark of these conditions is the build-up of incorrectly folded proteins within the brain, which results in neurological impairment and the onset of disease (4). The World Health Organization predicts that neurological conditions will rise to become the second most common cause of mortality among humans within the next two decades (5). The timely diagnosis for the majority of patients is impeded because of the insufficient presence of reliable biomarkers (6). Current interventions may decelerate the advancement of the illness; however, they fail to yield adequate outcomes and result in an unfavorable forecast. In order to surmount the constraints of present therapeutic approaches, innovative treatment methods must be devised to tackle neurodegenerative disorders (7).

Adult stem cells known as MSCs possess the ability to regenerate themselves and can differentiate into various cell lineages (8). The main origins from which MSCs are obtained consist of bone marrow (BM) (9), adipose tissue (ADI), as well as umbilical cord blood (UCB) (10). MSCs, which stem from the mesodermal layer, have been demonstrated in research to be capable of transdifferentiating into cells not originating from the mesoderm, including glial cells and neurons (11, 12). This is thought to be the best source for regrowing cells lost as a result of neurodegenerative illnesses. In addition, mesenchymal stem cells are easily obtained, isolated, and grown; they also show decreased immunogenicity and immunomodulatory potential, as well as immune system modulating qualities. As such, MSCs have a great deal of promise for the treatment of neurodegenerative diseases.

EVs are composed of a lipid bilayer and are produced and secreted by nearly every living cell (13, 14). These vesicles encapsulate various biologically active substances, including proteins, noncoding RNAs, and lipids (15, 16). EVs are acknowledged as one of the most potent means of communication between cells during both physiological and pathological events (17–19). Neurodegenerative disease-derived EVs and their contents can be a good response to the pathophysiologic state of the body (20). This characteristic endows them with the potential to serve as diagnostic instruments and as focal points for personalized treatment strategies. EVs engage with recipient cells by identifying and binding to specific receptors on the cell surface. A diverse range of neuronal subpopulations, including microglia, astrocytes, and Schwann cells, secrete these vesicles (21). With rapid advances in nanotechnology, enhanced EVs therapeutic capabilities have been developed, including targeted drug delivery. EVs are better biocompatible and less immunogenic than liposomes as carriers for synthetic drug delivery systems (22). EVs are functional pharmacokinetics-related proteins that contribute to their large biological distribution and higher cyclic retention. EVs’ surface molecules allow them to cross the blood-brain barrier, deliver cargo, and cause a response in the recipient cell. Due to these properties, EVs have considerable therapeutic potential in treating diseases of the central nervous system. In this review, we will present advances in MSC-derived extracellular vesicles in neurodegenerative disease models such as AD, PD, ALS, and HD. We will study the clinical results achieved by MSC-EVs therapy in various neurodegenerative disease models. We will also discuss the limitations of MSC-EVs therapy and the possibility of improving treatment efficiency to transition to clinical trials.

## Classification and biogenesis of EVs

In 2018, the International Society for Extracellular Vesicles (ISEV) revised its research criteria and gave EVs a new meaning. Particles in this category are a diverse mixture that typically range in size from 50 nm to 500 nm, and occasionally they can reach 1-10 μm. According to the ISEV, EVs should be named using a standardized method that would categorize them based on clear-cut, measurable characteristics such their size, unique chemical markers, and place of genesis in cells, among other things (14, 23, 24). ISEV proposes to categorize EVs by size into small EVs (sEVs) with diameters less than 200 nm and large EVs (lEVs) with diameters greater than 200 nm. The majority of contemporary research classifies extracellular vesicles into sEVs, microvesicles (MVs), and apoptotic bodies, primarily distinguishing them by their respective sizes.

**Figure 1:**
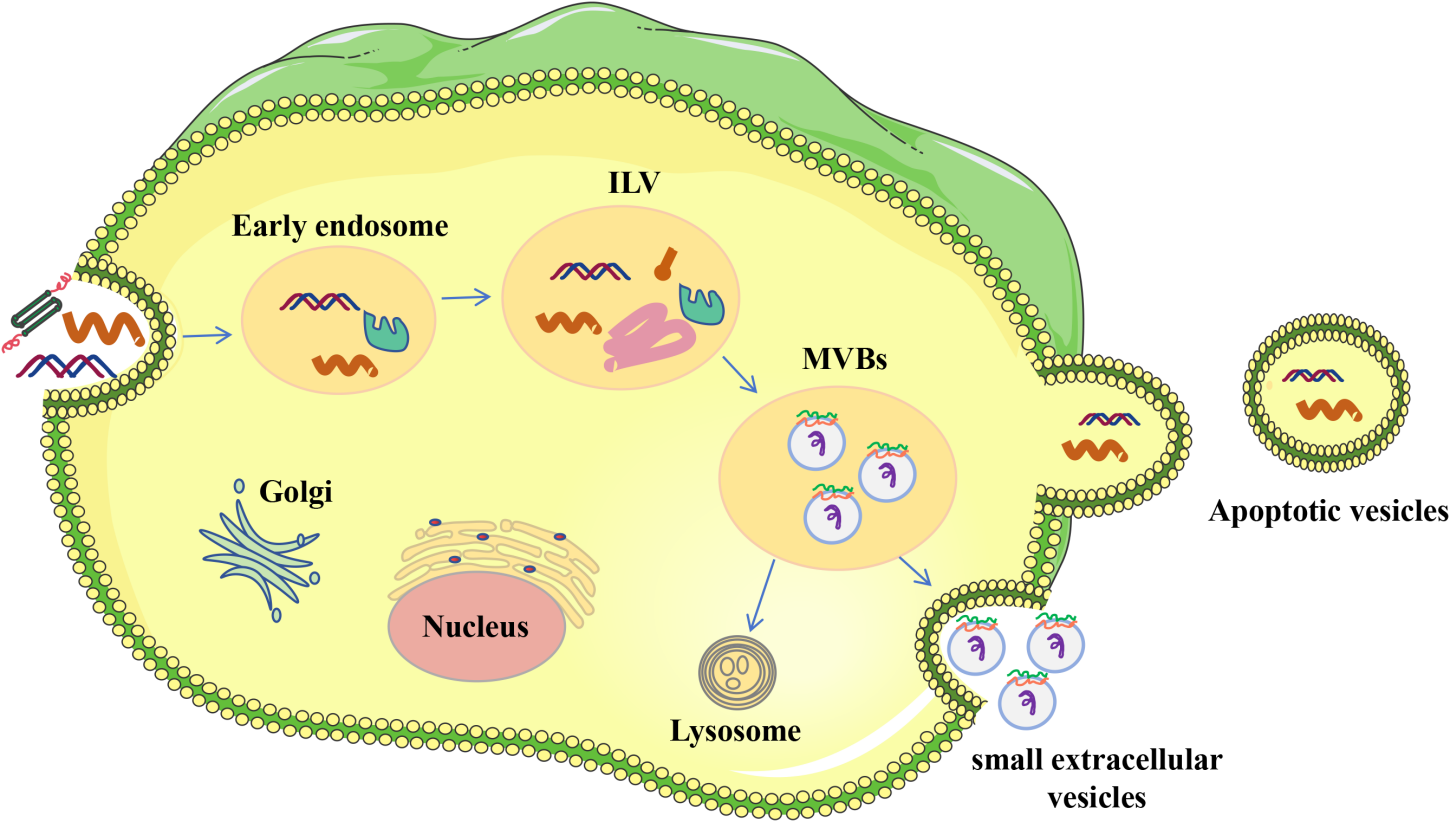
Origin of EVs. The process of endocytic generation produces sEVs. Donor cells’ plasma membranes first invaginate to create early endosomes, which later develop into late endosomes. Intracellular vesicles (ILVs) are formed when the membrane of early endosomes folds inward during this maturation phase. Multivesicular bodies (MVBs) are the term used to describe endosomes that include ILVs. ILVs are discharged into the extracellular milieu and are referred to as single-cell EVs when MVBs fuse with the plasma membrane.

sEVs represent the smallest of the EVs, having diameters that fall within the range of 50 to 150 nanometers (25). Typically, the formation of sEVs (refer to **Figure 1**) initiates as the cell membrane invaginates inwardly, creating endosomes within the cell (26). Cargo is divided into early endosomes inside the endosomal network, which later develop into late endosomes or multivesicular bodies. Intraluminal vesicles (ILVs) are rich in unique endosomal compartments known as late endosomes. The endosomal sorting complexes needed for transport (ESCRT) route and its machinery—which consists of four complexes, ranging from ESCRT-0 to ESCRT-III—assist in the synthesis of ILV (27). By enlisting hepatocyte growth-factor-regulated tyrosine kinase substrate to bind ubiquitinated cargo, the ESCRT-0 complex starts the process. Following ESCRT-II binding, ESCRT-II interacts with tumor susceptibility gene 101 (TSG101) to recruit ESCRT-I and start the budding of ILVs. ILV production is aided by the ESCRT-III subunits, which include Vps20, Snf7, Vps2, and Vps24. Lastly, Vps4 is used by ESCRT-III to separate the ILVs from the endosomal membrane (28–31). Once ILV formation is finished, the endosomes are effectively transformed into multivesicular bodies (MVBs). These MVBs can then fuse with the plasma membrane, leading to the release of ILVs from the cell as sEVs (32). MVBs may also merge with lysosomes, leading to the degradation of ILVs without the release of sEVs (33).

MVs are slightly larger in diameter than exosomes and are formed directly from cytoplasmic membrane outgrowth (34). The main process underlying the release of microvesicles (MVs) is contingent upon cytoskeletal modifications mediated by calpain, a protein whose activation is triggered by the influx of calcium (Ca2+) or its production within the endoplasmic reticulum (35, 36). Calcium ion concentration also plays a role in modifying the plasma membrane’s structure, suggesting that calcium ions alone can trigger both the production and the secretion of MVs (37). Certain studies have demonstrated that arrestin-domain-containing protein 1 (ARRDC1) is capable of recruiting ESCRT proteins, specifically TSG101 and Vps4, to the cell membrane, thereby triggering the initiation of membrane budding (38).

Apoptotic vesicles, ranging in size from 500 to 2000 nanometers, are sizable vesicles derived from apoptotic cells. They encapsulate cytoplasm, cellular organelles, and nuclear fragments (39). The leakage of the plasma membrane during apoptosis results in the generation of microvesicles, a form of apoptotic vesicles (40).

## EVs cargo

**Figure 2:**
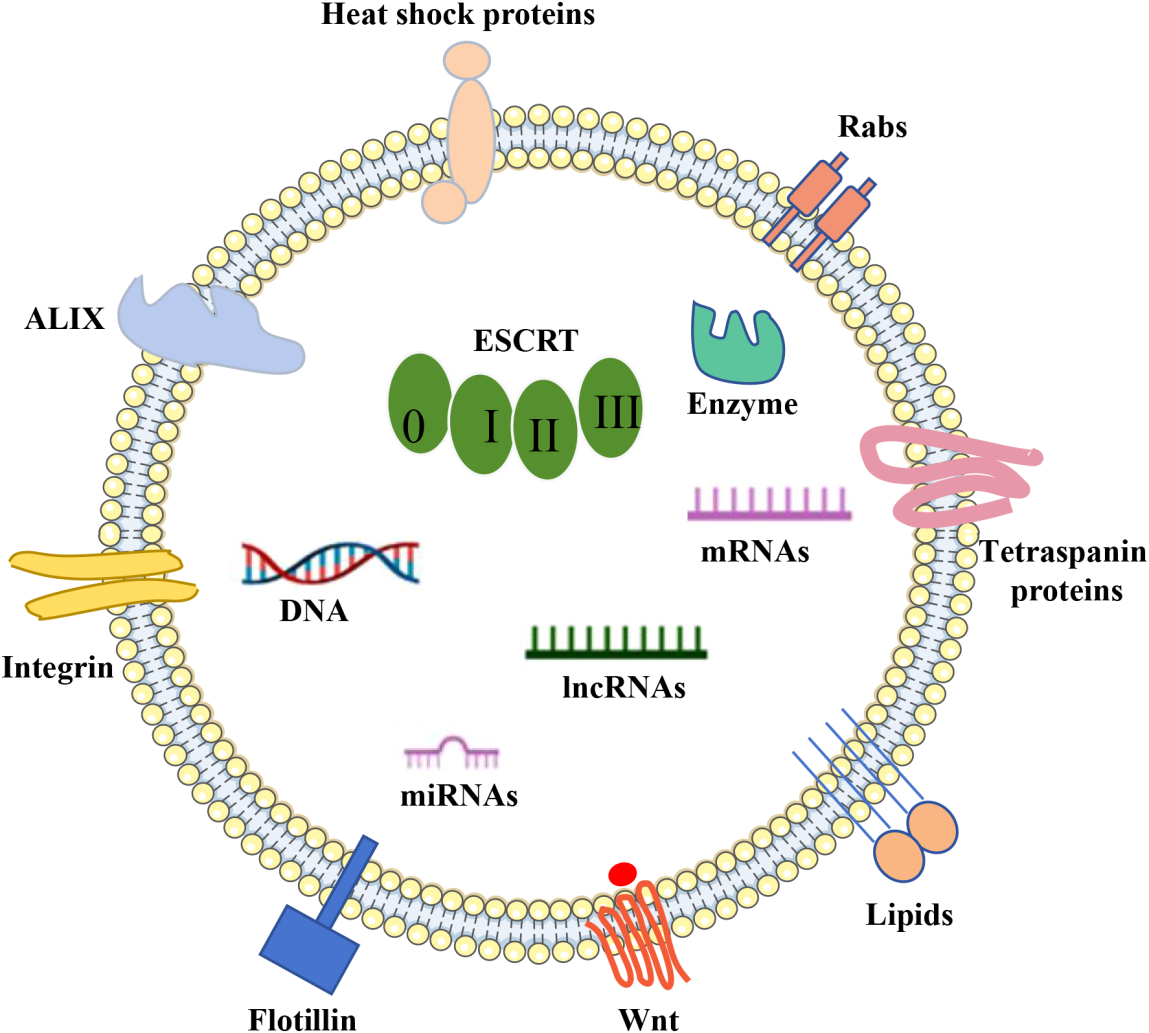
EVs cargo. DNA, RNA, lipids, metabolites, and a variety of membrane and intracellular proteins are all encased in a phospholipid bilayer that makes up extracellular vesicles (EVs). EV indicators include membrane and intracellular proteins include TSG101, Alix, CD63, CD9, and CD81.

The makeup of EVs is influenced by their cellular source and the process of their formation. These vesicles encapsulate a diverse array of biologically active substances, such as soluble proteins, nucleic acids, lipids, and metabolites (41). These molecules within the cargo are vital for cell-to-cell communication and are tasked with transporting a variety of signaling agents to their respective target cells (19) (**Figure 2**).

Proteins associated with the formation of EVs, like Alix and Tsg101, are acknowledged as cytoplasmic constituents of EVs and are utilized as general indicators of EVs (42). A further group includes tetraspanins, a family of membrane-bound proteins that have been demonstrated to influence cargo transportation in fibroblasts (43, 44). Several tetraspanin proteins, including CD63, CD9, and CD81, are commonly employed as specific EVs markers (45–47). Moreover, heat shock proteins are commonly found in EVs due to their involvement in protein stability (48, 49). Other exosomal proteins encompass nSMase 2 from the ceramide-dependent pathway (50–52), actin and flotillin, SNARE complexes, and Rab proteins (53).

EVs are repositories for various nucleic acids, including genomic DNA (54) and mitochondrial DNA (55), and RNA (mRNA, microRNA miRNA, lncRNA and circRNA) (30, 56). Among these, microRNAs are identified as one of the most prevalent RNA species within EVs (57, 58). These RNAs have been implicated in a wide array of biological processes, including the neurogenesis aspect of neurodegenerative conditions like PD and AD (59–62). For instance, EVs secreted by hypoxia-conditioned mesenchymal stromal cells have been found to enhance cognitive function in APP/PS1 mice by addressing synaptic impairment and modulating inflammatory reactions (63). EVs miRNAs are encapsulated in lipid bilayers, protected from nuclease degradation, and are extremely stable in body fluids (e.g., blood, cerebrospinal fluid). miRNAs enter the peripheral circulation and offer the possibility of early diagnosis of neurodegenerative diseases. A study found that the level of miR-132 in plasma EVs of AD patients was significantly reduced, which can be used as an early diagnostic marker (64).

Lipids are integral to preserving the structural integrity of EVs, including their membrane dynamics, secretion, and internalization processes, as well as their functional role within the vesicles (65). These lipids are not only pivotal in the synthesis and uptake of EVs but also serve as a class of bioactive molecules that participate in diverse biological functions, such as immune system surveillance, modulation of the tumor microenvironment, and the regulation of inflammatory responses (66, 67).

## Mechanism of EVs in neurological diseases

A common feature of neurodegenerative diseases is the misfolding, aggregation, and accumulation of pathologic amyloid proteins within or outside brain cells. EVs are novel and important carriers of signaling molecules *in vivo*, and a growing body of literature highlights the important role of EVs in the intercellular transmission of pathogenic protein aggregates, thus contributing to the understanding of the pathology and clinical progression of neurodegenerative diseases (68). These EVs can carry and protect a range of proteins, lipids and nucleic acids from degradation in the extracellular space. Recently, several studies have shed light on the physiological role of EVs in neurodegenerative diseases, including the regulation of glutamatergic synaptic activity during nerve cell development. Astrocytes maintain brain homeostasis by internalizing miR-124 from microglia-derived EVs to regulate levels of glutamate transporter 1 and glutamate uptake (69). Another study showed that stimulation of 5-hydroxytryptamine receptors increased the release of insulin-degrading enzymes from microglia via EV, which were able to degrade the neurotoxic peptide amyloid β (70). EVs from human bone marrow-derived endothelial progenitor cells have the potential to mend damaged microvessels in the central nervous system (CNS) of symptomatic SOD1-G93A mutant mice (71). Additionally, Guo and colleagues discovered that microglial exosomes facilitate the transmission of α-synuclein (α-syn) between cells, contributing to neurodegeneration in the substantia nigra and striatum, which is a significant factor in PD pathogenesis (72). Thus, EVs not only play an important role in the development of neurodegenerative diseases, neuroprotection, repair and further regulation of neuronal activity, but also participate in the onset and progression of their diseases. EVs exert neuroprotective effects through multiple mechanisms: Anti-inflammatory effects; EVs inhibit neuroinflammation by delivering anti-inflammatory molecules (e.g., miRNAs and cytokines) and protect neurons from inflammatory damage. For example, miR-124 carried by EVs inhibits microglia activation and reduces the release of inflammatory mediators (73). Antioxidant effects; EVs reduce oxidative stress by delivering antioxidant molecules (e.g., superoxide dismutase and glutathione) that protect neurons from oxidative damage (51). Promote neuronal survival; EVs promote neuronal survival and growth by delivering neurotrophic factors such as brain-derived neurotrophic factor and glial cell-derived neurotrophic factor (74). Promoting synaptic plasticity; EVs promote synaptic plasticity and enhance neuronal function by delivering synapse-associated proteins and miRNAs. For example, synapsin I carried by EVs promotes the release of synaptic vesicles and enhances synaptic transmission (75).

In addition to their application in treating various neurological conditions, this mechanism has unlocked a plethora of potential applications (76). EVs are pivotal in providing insights into CNS functions and pathologies due to their critical role in intercellular communication. EVs carry molecules (e.g., proteins, lipids, and nucleic acids) that bind to receptors on the surface of neurons, triggering signaling that affects neuronal activity and function. For example, integrins on the surface of EVs bind to extracellular matrix proteins on neurons, affecting neuronal survival and synaptic plasticity (77). EVs carry ligands (e.g., Epidermal Growth Factor, Fibroblast Growth Factor)) that bind to receptor tyrosine kinases on neurons, activating downstream signaling pathways that regulate neuronal growth and differentiation (43). In addition, It has been proposed that EVs could serve as drug delivery systems across the blood-brain barrier (BBB), enabling targeted therapy within the CNS (78). MSC-EVs have been demonstrated to alleviate neurological diseases like Parkinson’s, Alzheimer’s, strokes, and amyotrophic lateral sclerosis, despite coming from a variety of sources and being engineered to elicit therapeutic effects in various diseases. MSC-EVs are attractive options for cell-based therapeutic approaches due to their special qualities.

## MSC-EVs and AD

The most common and well-known kind of dementia is AD, which has been the focus of much research but is still not fully understood. This neurodegenerative illness affects several facets of brain function, including cognitive capacities, personality qualities, the structure of mental processes, and behavioral patterns. It is distinguished by its progressive and irreversible nature (79). Numerous studies have pointed to the buildup of amyloid beta peptide, a key constituent of amyloid plaques, within neurons as the primary factor driving the onset of AD pathology (80). Research indicates that the primary location for the synthesis of amyloid beta (Aβ) by neurons is within the MVBs (81). These bodies are made of internal vesicles (ILVs), which have been shown in numerous studies to have two distinct fates: either they are released into the extracellular environment as exosomes through a process of fusion with the plasma membrane, or they are targeted for degradation within the lysosomal interior (82). These findings underscore the significance of exosomes in the progression of Alzheimer’s disease, particularly with regard to the dissemination of Aβ aggregates (83). The levels of Aβ in AD brains can be markedly decreased by interfering with the pathways that generate Aβ, as well as by introducing agents to the brain that facilitate the degradation of Aβ peptides. In this context, MSC-EVs could prove instrumental. The amyloid-β precursor protein (APP) is cleaved by β-and γ-secretases, resulting in the production of neurotoxic Aβ peptides in AD brains. Under these circumstances, MSC-EVs can be engineered to release vesicles containing siRNA that targets β-and γ-secretases, thereby significantly diminishing their activity. According to a recent study, EVs that include proteases may act as a conduit for information between the brain and the body’s peripheral organs. Extracellular vesicles produced from plasma have been shown to accelerate the onset of Alzheimer’s disease in transgenic mice by cleaving substrates such as amyloid precursor protein (APP) in target neurons. According to this research, plasma EVs may be harmful in the development of AD (84, 85). Furthermore, exosomes facilitate the absorption of extracellular Aβ plaque by microglial cells (51, 86). Curiously, a significant number of research efforts have focused on the potential therapeutic use of EVs secreted by MSCs, as outlined in **Table 1**.

**Table 1:** The roles of MSC-EVs in Alzheimer’s disease.

Disease	Source of EVs	Route of administration	Outcomes	References
Alzheimer's disease	Murine neuroblastoma Neuro2a (N2a) cells	Stereotactic administration	Reduced amyloid-β peptide (Aβ) levels and improved Aβ pathology.	(86)
	Wharton’s jelly mesenchymal stem cells	Not reported	Increases the resistance of hippocampal neurons to damage caused by Aβ	(87)
	Mesenchymal stem cells	Stereotactic administration	stimulated neurogenesis in the subventricular zone and alleviated beta amyloid 1−42-induced cognitive impairment, and these effects are similar to those shown in the mesenchymal stem cells.	(88)
	Bone marrow mesenchymal stem cells	Cocultures of hippocampal neurons and MSC-EVs	Protection of neurons against amyloid-β peptide-induced oxidative stress and synaptic damage.	(89)
	Bone marrow mesenchymal stem cells	Intranasal administration	Regulation of microglia phenotype and dendritic spine integrity.	(90)
	Neural stem cells	Bilateral ventricles	Enhanced mitochondrial function, SIRT1 activation, synaptic activity, decreased inflammatory response, and rescued cognitive deficits in AD	(91)

According to studies, MSC-EVs have been observed to mitigate neuronal injuries and restore synaptic function (63). Within this framework, MSC-EVs have demonstrated the ability to enhance neuroprotection and encourage neuroregeneration (87, 88). De Godoy et al. have proposed many plausible pathways via which MSC-EVs and MSC transplantation might provide neuroprotective benefits against neuronal damage caused by Aβ. These include the paracrine effects of the extracellular release of inflammatory factors and anti-inflammatory cytokines like IL-6, IL-10, and VEGF, the reduction of extracellular Aβ oligomer levels because of the high endocytic capacity of MSCs, and the secretion of EVs containing antioxidant enzymes like catalase (89). likewise, in order to explore the immune-regulatory properties, EVs released from primed MSCs were introduced intraperitoneally (ITI) into a triple transgenic model of Alzheimer’s disease (3xTg AD). The results of the study showed that this therapy caused COX2 and IDO to be overexpressed, which in turn decreased the synthesis of IL-6 and IL-1β. On the other hand, it enhanced IL-10 production, which supported the M2 macrophage phenotype (90). Perets et al. found that MSC-EVs specifically targeted and accumulated in pathologically associated brain regions in mouse models of AD within 96 hours after administration, whereas in healthy controls, they showed diffuse migration patterns and clearance at 24 hours. This suggests that neuroinflammatory signaling in the pathological brain is highly correlated with MSC-EVs accumulation, suggesting that the homing mechanism is inflammatory driven (91). This discovery could significantly promote the application of MSC-EVs in the treatment and targeted drug delivery of AD.

In a parallel vein, a multitude of *in vivo* experiments have indicated that MSC-EVs alleviate symptoms associated with AD. In 2018, Cui and his team investigated the recovery from cognitive impairment in an APP/PS1 mouse model of AD through the use of exosomes derived from mesenchymal stromal cells that had been preconditioned under hypoxic conditions. While both MSCs and hypoxia-preconditioned MSC-derived exosomes reduced both intracellular and extracellular Aβ oligomer deposits, the hypoxia-preconditioned MSC-derived exosomes showed a more pronounced effect in improving learning and memory deficits. This was achieved by reducing serum levels of pro-inflammatory cytokines (IL-1β and TNF-α) and concurrently increasing the levels of anti-inflammatory cytokines (IL-4 and IL-10). Additionally, the exosomes reduced inflammatory responses by inhibiting the function of astrocytes and microglia. Furthermore, the activated levels of STAT3 and NF-κB in the brains of transgenic mice treated with hypoxia-preconditioned MSC-derived exosomes were found to be decreased (63). A recent study by Li provides insight into the potential of mesenchymal stem cell-derived EVs in alleviating cognitive impairment in mouse models of AD. The main findings of this study showed that factors related to mitochondrial function, such as SIRT1 and synaptic proteins, were upregulated, while markers of oxidative damage, inflammatory cytokines, and microglia activity were significantly reduced compared to the control group (92). A separate study employed heat-shock-treated neural stem cell-derived exosomes in the treatment of a mouse model of AD, resulting in the restoration of cognitive impairment and enhancement of motor skills (93). Building on this encouraging evidence, the deployment of mesenchymal stem cell-derived MSC-EVs in the treatment of AD seems to offer a promising and innovative approach to address intractable medical conditions.

## MSC-EVs and PD

An analogous situation is foreseen for PD, the second most prevalent chronic neurodegenerative condition globally (94), marked by the degeneration of dopamine-producing neurons coupled with the accumulation of α-synuclein protein clumps within the neurons’ internal architecture, leading to a reduction in dopamine synthesis across multiple brain networks (95).

EVs have attracted a lot of interest as a crucial component in the etiology of PD. Studies conducted as early as 2010 have shown that EVs are able to transport α-syn, which has helped to advance the extracellular seeding theory. Additionally, studies have shown that EVs from PD patients’ cerebrospinal fluid can cause α-syn aggregation in recipient cells, which may aid in the pathological advancement of the illness (96). The potential of MSC-EVs as a therapeutic approach appears promising, albeit in its preliminary stages. In PD, key factors contributing to pathology include mitochondrial dysfunction, impairments in protein degradation pathways such as the ubiquitin–proteasome system, and disruptions in the autophagy-lysosomal pathway (97). The utilization of MSC-EVs in PD is detailed in **Table 2**.

**Table 2:** The role of MSC-EVs in Parkinson’s disease.

Disease	Source of EVs	Route of administration	Outcomes	References
Parkinson’s disease	Human exfoliated deciduous teeth stem cells	Not reported	Inhibition the apoptosis-induced by (6-OHDA) in human dopaminergic neurons	(99)
	Human exfoliated deciduous teeth stem cells	Intranasal administration	normalizes tyrosine hydroxylase expression in the substantia nigra and striatum of the (6-OHDA)-treated rats	(100)
	Human umbilical cord mesenchymal stem cells	Tail vein injections	Neuroprotection of dopaminergic neuron in substantia nigra and upregulation of dopamine levels in striatum	(101)
	Bone marrow mesenchymal stem cells	Tail vein injections	Reduction in α-syn aggregates and functional recovery	(102)
	Bone marrow mesenchymal stem cells	Tail vein injections	Regulate neurite outgrowth by transfer of the miR-133b	(108)
	Bone marrow mesenchymal stem cells	Not reported	Stimulation of oligodendrogenesis and improving neuronal function	(109)

A transgenic rat model of PD was used to study the effects of human mesenchymal stem cell (hMSC) conditioned medium. The results show that the hMSC-secretome has a promising role in increasing the number of dopaminergic neurons, helping to partially recover motor impairments, and reducing histological manifestations of the disease (98). The importance of these investigations stems from the fact that EVs play a vital role in a cell’s secretome and, as a result, the EVs that a cell secretes affect certain outcomes. For example, Jarmalavičiőtė et al. investigated the neuroregenerative potential of exosomes and microvesicles generated by human exfoliated deciduous tooth (SHED) stem cells on dopaminergic neurons. Their results showed that exosomes released by SHEDs, but not microvesicles, might inhibit 6-OHDA-induced apoptosis in human dopaminergic neurons. In their conclusion, they suggested using SHED exosomes as a therapeutic method to treat PD (99). Later work by the same group used EVs derived from human exfoliated deciduous teeth (SHEDs) stem cells to reduce motor symptoms in 6-OHDA-induced unilateral lesion models of PD. The results demonstrated that the EVs improved motor functions in addition to stopping the 6-OHDA-induced gait abnormalities. The restoration of striatal tyrosine hydroxylase (TH) activity and levels was credited with this improvement (100). Chen and colleagues discovered that EVs originating from mesenchymal stem cells can mend a Parkinson’s disease model by stimulating autophagy (101). Peng and colleagues created a self-guided nanocarrier named PR-EXO/PP@Cur, merging therapeutic MSC-EVs with curcumin. This integration enhances the functional restoration of neurons and lessens neuroinflammatory responses by diminishing the levels of α-synuclein aggregates (102). In Parkinson’s disease, as with other neurological disorders, the profiling of miRNA expression is viewed as an effective instrument for both diagnostic and therapeutic objectives (103). For example, miR-433 and miR-16-1 are involved in Parkinson’s disease-related pathological mechanisms that elevate α-synuclein levels (104). Furthermore, the reduction in miR-34b/c levels and the increase in miR-494 and miR-4639-5p expression exert opposing influences on DJ-1 protein levels, with the former negatively affecting and the latter positively impacting its expression. DJ-1 is known as a protector against mitochondrial oxidative damage (105, 106). Moreover, MSC-EVs facilitate neural differentiation by conveying both endogenous and exogenous microRNAs. To illustrate, Lee and colleagues verified the differentiation phenotype in human neural progenitor cells (NPCs) and observed an increase in glutamate transporter expression in both NPCs and astrocytes following the delivery of two specific exogenous microRNAs, miR-124 and miR-145, via MSC-EVs (107). In a separate instance, studies have noted that while miR-133b is markedly decreased in individuals with PD, it is abundant within MSC-EVs. Both *in vitro* and *in vivo* experiments demonstrated that the delivery of miR-133b via MSC-EVs promotes neuronal growth (108). Xin and colleagues discovered that the MiR-17-92 cluster within EVs boosts neuroplasticity and aids in functional recovery following stroke in rats (109). Although the research is somewhat limited, the current results have clearly shown the advantageous impacts of various stem cell types in the management of Parkinson’s disease, which is largely attributed to the payload of their inherent EVs.

## MSC-EVs and ALS

ALS is a intricate, advancing neurodegenerative disorder. The disease’s onset is linked to a multitude of pathological processes, such as mitochondrial impairment, oxidative stress, and axonal destruction (110), which result in the deterioration and death of neuronal cells. ALS predominantly affects males (111) and progresses swiftly. It is marked by the deterioration of both upper and lower motor neurons (MNs) within the brain and spinal cord, causing incremental muscle wasting and limb frailty. The majority of patients succumb to respiratory complications within a few years following the emergence of symptoms (111). Current medications merely alleviate symptoms. Effective treatment of ALS could be achieved if the lifespan of MNs could be extended. MSC-based therapies may pave the way for more potent treatments for ALS.

Over time, several molecular targets have been suggested to play a role in the development of ALS, and a variety of proteins that are encoded by genes associated with the pathogenesis of ALS have been identified. Recent research suggests that a large number of these proteins are either expressed differently or are found within EVs and can move between neurons and glial cells in different parts of the brain, facilitating the dissemination and propagation of EVs.

Among these proteins are SOD1 (112), TDP-43 (113), Fused in sarcoma (FUS) (114), and Dipeptide repeating proteins (DPRs) (115). Provenzano et al. found that EVs from MSCs attenuated the pathological phenotype and neurotoxicity in human astrocytes (iAstrocytes) derived from inducible neural progenitor cells (iNPCs) of ALS patients as well as in astrocytes extracted from the spinal cords of symptomatic SOD1G93A mice. The neurotoxic effects of mouse and human ALS astrocytes on motor neurons were reversed by *in vitro* exposure to EVs (116). EVs notably reduced the pathological traits and neuroinflammation in SOD1G93A astrocytes. Lee and associates demonstrated the differentiation of neural stem cells from (SOD1(G93A)) transgenic ALS mice following treatment with EVs derived from adipose-derived stem cells. The analysis revealed a decrease in cytosolic SOD1 aggregates and an improvement in mitochondrial protein markers, such as the phospho-CREB/CREB ratio and PGC-1α (117). In a parallel study, the protective influence of EVs from adipose-derived mesenchymal stem cells against oxidative damage was confirmed in an *in vitro* model expressing ALS mutations (118).

Intriguingly, every study has uniformly reported enhancements in physical prowess without any notable detrimental side effects (119, 120). In particular, Crose’s research noted that a patient, after receiving treatment with EVs derived from human bone marrow mesenchymal stem cells (BM-MSCs), was able to ambulate with minimal support for a distance of 25–50 feet, following a six-month period of being unable to walk (119). Likewise, Ueda and associates documented an augmented range of motion in the limbs (120). Additional enhancements included better speech and increased strength, a decrease in muscle spasms (and consequently the related discomfort) (119), as well as a slowdown in the decline of respiratory function (120). These benefits are likely associated with the shielding effect of the MSC-secretome/EVs on limbal motor neurons, the neuromuscular junction, and muscle tissue, along with protection against inflammation, as indicated by a reduction in glial cell activation. Moreover, enhancements in sleep quality were also noted (119).

## MSC-EVs and HD

HD is mainly identified by involuntary dance-like movements, along with emotional and cognitive impairments, which ultimately result in fatality (121). There are several mechanisms of action of MSC-EVs in HD: 1 Immunomodulation; Studies have shown that MSC-EVs are able to modulate the immune response and attenuate neuroinflammation through multiple mechanisms, which is crucial for patients with HD. MSC-EVs are capable of releasing a variety of factors that have immunomodulatory functions, such as cytokines and growth factors, which in turn inhibit the release of over-activated microglial cells and inflammatory mediators, thereby attenuating nerve damage and apoptosis (122). 2 Promote neuroprotection and regeneration; in an animal model of HD, the application of MSC-EVs significantly improved neurological function, reduced neuronal apoptosis, and promoted regeneration of damaged nerves (123). 3 Improvement of cell metabolism and function; MSC-EVs can improve the metabolic state of neurons and enhance their function by regulating energy metabolic pathways. For example, MSC-EVs can promote the function of mitochondria and improve cellular ATP production, which in turn improves the viability and function of neuronal cells (124).HD currently has no known cure; palliative care is the only treatment available. Like in other neurodegenerative diseases, EVs allow huntingtin proteins with polyglutamine expansions to spread to nearby cells (125). Therefore, EVs play a crucial role in the pathophysiology of HD. Many MSC-EVs-based therapy approaches have been evaluated for the management of HD (126).

Lee and colleagues conducted research in this area and observed that exosomes from adipose-derived mesenchymal stem cells can modulate harmful properties in HD cell models (127). Additionally, the same research group investigated the delivery of miR-124 through exosomes to the striatum of R6/2 HD transgenic mice. Despite observing a decrease in the intracellular expression of the miR-124 targeted gene, REST, the effects on the mice’s behavior were minimal (128). Studies have shown that MSC-EVs have particular effects on HD. *In vitro* analysis has revealed that MSC-EVs can constrain motor function and striatal atrophy in a rat model of HD (129). In their study, Ebrahimi and colleagues showed that the release of GDNF and vascular endothelial growth factor (VEGF) from MSCs had a positive effect on motor coordination and muscle functions in animal models of HD (130).

## MSC-EVs in clinical trials

Over 200 clinical trials of exosomes or extracellular vesicles treatments are listed on the clinicaltrials.gov website. Nine of these studies use MSC-derived exosomes. A number of pharmaceutical companies as well as the academic community have expressed interest in EVs-based therapeutics. The number of clinical trials for EVs-based therapeutics in humans is rapidly increasing, and more are currently being conducted (131). The following clinical trials are currently registered in the field of neurological disorders: A Phase I/II clinical trial study of adipose MSC-EVs administered by nasal drip into subjects with Alzheimer’s disease has been evaluated for safety and efficacy(NCT04388982). Another study demonstrated the great potential use of different groups (Autologous/allogenic UC-MSCs + A-MSC-secretome) implanted in Multiple System Atrophy patients (NCT04876326). Another study evaluating the improvement of patients with acute ischemic stroke treated with MSC -EVs fully demonstrated its safety and efficacy (NCT03384433). In all three trials, MSCS provided EVs, although their tissue sources were different. These representative clinical trials of EV-based therapies for human neurodegenerative diseases will yield exciting results (132). Despite impressive preclinical results in both clinical and biochemical parameters, the use of MSC-derived EVs in clinical trials remains limited.

## Advantages and limitations of MSC-EVs therapy

MSC-EVs offer a promising alternative to cell therapy because they can provide the beneficial effects of MSCs. MSC-EVs have the following advantages over cellular therapies in clinical applications (1): Safety. MSC transplantation may be rejected by the host immune system, and MSC-EVs are less immunogenic. On the other hand, MSC transplantation carries the risk that the cells will differentiate in an undesirable manner, potentially transforming into malignant cells and forming tumors. Due to the lack of self-replicating ability, EVs do not have the potential to generate tumors (2). Target tissues. Unlike natural MSCs, EVs are able to cross the blood-brain barrier via cytosis and exert direct effects in the brain. EVs crossing of the BBB in a bidirectional manner between the bloodstream and brain parenchyma remains poorly understood. There are several mechanisms: receptor-mediated transport, EVs express a variety of receptors and ligands on the surface, which can bind to the receptors on the surface of BBB endothelial cells, triggering endocytosis, thus realizing transmembrane transport (133). Adsorption-mediated transport, cationic molecules on the surface of EVs (e.g., phosphatidylserine) can interact with anionic molecules on the surface of BBB endothelial cells, triggering adsorption-mediated transport (134), and EVs are able to pass through the BBB endothelial cells directly into the brain tissue through intercellular communication mechanisms (e.g., Tunneling Nanotubes) (135). (3). Versatility. One advantage of EVs is that they can be modified in multiple ways to increase their therapeutic potential. One strategy is to enrich them in a microRNA or protein that has a beneficial effect. Another strategy is to modify membrane proteins on the vesicle surface to increase their specificity for specific target tissues.

Although MSCs-EVs can be manipulated to alleviate some limitations, MSC-EVs treatment faces additional challenges. Because living cell treatments have the potential to trigger an immune response or cause tumor growth, they may not have the natural implantation capabilities of normal cells (136). Furthermore, standardizing and improving EV production is essential to overcoming the current barriers to the development of medicines based on EVs. Furthermore, further investigation is required to improve our comprehension of their mechanics.Technical issues that impact the production processes, such as determining the best cellular source, culture and storage conditions, cell type variability, and phenotypic instability during cell passaging, are mostly to blame for a number of variations (137). These discrepancies are also a result of inconsistent and standardised techniques for vesicle extraction and characterisation. The constraints of current technology have made it difficult to identify, isolate, and analyze EVs, and research done in the last 10 years has frequently been tainted by artifacts. In 2018, the Minimal Information for Studies of Extracellular Vesicles (MISEV) rules were modified and suggested by the International Society for Extracellular Vesicles (ISEV) in response to concerns over process uniformity. The goal of these updates is to improve and guarantee the caliber of EV research. Natural EVs show potential in treating neurodegenerative illnesses; nevertheless, their short half-life, poor targeting precision, quick clearance upon injection, and small payload restrict their therapeutic use. Studies conducted on humans and preclinical models have shown that following systemic injection, EV levels in the blood drop off quickly, and that EVs tend to concentrate in the liver, spleen, and lungs for around ten days. Additionally, the clearing of macrophages and microglia reduces the duration of EV circulation (138).

## Conclusions

All things considered, the research that has been published supports the notion that MSC-EVs may offer a new kind of treatment for neurodegenerative diseases. Because EVs may cross the blood-brain barrier and enter the damaged parts of the brain, this is a significant benefit of using them in neurodegenerative disease treatment. Furthermore, these EVs have the ability to transport and carry certain molecules, such as microRNAs, which are essential for controlling gene expression. Neuroprotection can be improved and gene expression patterns influenced by EVs carrying these microRNAs to brain cells. Moreover, MSC-EVs can be used as delivery vehicles for molecules or therapeutic agents, allowing for targeted delivery to the afflicted brain areas. By using a targeted delivery approach, the therapeutic impact of the chemicals delivered is increased and the probability of negative effects is decreased. While there are still many obstacles to overcome, MSC-EVs are a novel and exciting treatment option for neurodegenerative illnesses.
